# Associations of Anthropometric Factors with KRAS and BRAF Mutation Status of Primary Colorectal Cancer in Men and Women: A Cohort Study

**DOI:** 10.1371/journal.pone.0098964

**Published:** 2014-06-11

**Authors:** Jenny Brändstedt, Sakarias Wangefjord, Björn Nodin, Jakob Eberhard, Magnus Sundström, Jonas Manjer, Karin Jirström

**Affiliations:** 1 Department of Clinical Sciences, Division of Oncology and Pathology, Lund University, Skåne University Hospital, Lund, Sweden; 2 Department of Surgery, Skåne University Hospital, Malmö, Sweden; 3 Department of Immunology, Genetics and Pathology, Uppsala University, Uppsala, Sweden; 4 Department of Clinical Sciences, Malmö, Lund University, Skåne University Hospital, Malmö, Sweden; 5 Department of Plastic Surgery, Skåne University Hospital, Malmö, Sweden; University of Bari & Consorzio Mario Negri Sud, Italy

## Abstract

Obesity is a well-established risk factor for colorectal cancer (CRC), and accumulating evidence suggests a differential influence of sex and anthropometric factors on the molecular carcinogenesis of the disease. The aim of the present study was to investigate the relationship between height, weight, bodyfat percentage, waist- and hip circumference, waist-hip ratio (WHR), body mass index (BMI) and CRC risk according to KRAS and BRAF mutation status of the tumours, with particular reference to potential sex differences. KRAS and BRAF mutations were analysed by pyrosequencing in tumours from 494 incident CRC cases in the Malmö Diet and Cancer Study. Hazard ratios of CRC risk according to anthropometric factors and mutation status were calculated using multivariate Cox regression models. While all anthropometric measures except height were associated with an increased risk of KRAS-mutated tumours, only BMI was associated with an increased risk of KRAS wild type tumours overall. High weight, hip, waist, WHR and BMI were associated with an increased risk of BRAF wild type tumours, but none of the anthropometric factors were associated with risk of BRAF-mutated CRC, neither in the overall nor in the sex-stratified analysis. In men, several anthropometric measures were associated with both KRAS-mutated and KRAS wild type tumours. In women, only a high WHR was significantly associated with an increased risk of KRAS-mutated CRC. A significant interaction was found between sex and BMI with respect to risk of KRAS-mutated tumours. In men, all anthropometric factors except height were associated with an increased risk of BRAF wild type tumours, whereas in women, only bodyfat percentage was associated with an increased risk of BRAF wild type tumours. The results from this prospective cohort study further support an influence of sex and lifestyle factors on different pathways of colorectal carcinogenesis, defined by KRAS and BRAF mutation status of the tumours.

## Introduction

It is well established that body size influences risk of colorectal cancer (CRC). It has however been less investigated whether this risk differs according to molecular subsets of the disease. Colorectal carcinogenesis is a multistep process driven by accumulation of several genetic alterations, including chromosomal abnormalities, gene mutations, and epigenetic modifications involving regulation of proliferation, differentiation, apoptosis, and angiogenesis [1], [2]. At least three distinct pathogenetic pathways have been identified, i.e. the chromosomal instability (CIN), microsatellite instability (MSI), and CpG island methylator phenotype (CIMP) pathways [3], [4].

Somatic mutations in the KRAS (v-Ki-ras2 Kirsten rat sarcoma viral oncogene homolog) oncogene are identified in 30–40% of sporadic CRC and these mutations occur early in the carcinogenetic process [5], [6]. While KRAS mutation predicts non-responsiveness to epidermal growth factor receptor (EGFR)-targeting agents, the prognostic relevance of KRAS mutations still remains controversial [7]–[9].

Recent studies suggest that BRAF (v-Raf murine sarcoma viral oncogene homolog B1) mutations occur in 10–20% of sporadic CRC [10]. BRAF mutations are more frequent in women, in right-sided tumours and are more often associated with lower differentiation grade, mucinous histology and, subsequently, a poor prognosis [9], [11]–[16].

KRAS and BRAF mutations are nearly always mutually exclusive, and BRAF mutations are relatively rare in conventional adenomas, but closely associated with the CIMP pathway, which is found in 70–80% of all dysplastic serrated lesions of the right colon, predominantly in women [6].

Body weight and BMI are the most commonly used anthropometric measurements in studies on the associations of obesity and CRC risk, the majority of which have shown a positive relationship between BMI and risk of CRC in men, but weak or no associations in women [17], [18]. The more precise mechanisms behind the discrepancy between men and women regarding the association of body size with CRC risk remain unclear, and may be related to differences in fat distribution between the sexes. It is also possible that evaluation of risk in relation to specific molecular tumour markers may help clarify these associations. Since KRAS and BRAF mutations are thought to occur early in colorectal carcinogenesis [5], [6], it seems biologically plausible that exposures such as obesity might modulate CRC risk differentially according to KRAS and BRAF mutation status of the tumours [19], [20].

Hence, the aim of this prospective cohort study was to investigate the associations of obesity, measured as several anthropometric factors, with CRC risk according to KRAS and BRAF mutation status of the tumours, overall, and with particular reference to potential sex differences.

## Methods

### Study group

Between 1991 and 1996, a total number of 28 098 individuals; 11 063 (39,4%) men and 17 035 (60,6%) women, between 44–74 years were enrolled in the prospective, population-based cohort study Malmö Diet and Cancer Study (MDCS), from a background population of 74 138 [21]. All participants completed the baseline examination, which included a questionnaire, anthropometric measurements and a dietary assessment. The questionnaire covered information on physical activity, use of tobacco and alcohol, heredity, socio-economic factors, education, occupation, previous and current disease and current medication. In addition, blood samples were collected and stored in −80°C. Follow-up is performed annually by record-linkage to national registries for cancer and cause of death.

Cases were identified from the Swedish Cancer Registry up until 31 December 2007, and from The Southern Swedish Regional Tumour Registry for the period of 1 January to 31 December 2008.

Until end of follow-up 31 December 2008, 584 incident cases of CRC had been registered in the MDCS. Eight tumours were re-classified as intramucosal cancer, and these were not included as cases, but did, however, contribute with person-years in all analyses. A total number of 181 cases were diagnosed with CRC before baseline examination, i.e. prevalent colorectal cancers, and therefore excluded from the study.

All tumours with available slides or paraffin blocks were histopathologically re-evaluated by a senior pathologist (KJ) on haematoxylin and eosin-stained slides. Histopathological, clinical and treatment data were obtained from the clinical and/or pathology records. Information on vital status and cause of death was obtained from the Swedish Cause of Death Registry up until 31 December 2009. Patient and tumour characteristics of the cohort have been described in detail previously [22]–[24]. Ethical permissions for the MDCS (Ref. 51/90), and the present study (Ref. 530/2008), were obtained from the Ethics Committee at Lund University.

### Anthropometric measurements

At baseline examination, weight (multiples of 0.1 kg) and height (to the nearest 0.005 m) were measured and body mass index (BMI) was calculated as kg/m^2^. Waist circumference was measured at the midpoint between the lower ribs and the iliac crest, and for hip circumference the level of greatest lateral extension was used. These measurements were estimated to the nearest 0.01 m. The waist and hip circumferences of each participant were used to calculate waist-hip ratio (WHR; cm/cm) as an additional measure of fat distribution. All anthropometric measurements were performed by a trained nurse. Body composition was estimated using a single frequency bio-impedance methodology (BIA 103, RLJ-systems, Detroit, MI, USA) with tetra polar electrode placement and subjects in a supine position. Lean body mass and fat mass were determined and served to calculate body fat percentage. The BIA method has previously been validated in Swedish middle-aged and elderly adults [25].

### Pyrosequencing analysis of KRAS and BRAF mutations

The PyroMark Q24 system (Qiagen GmbH, Hilden, Germany) was used for pyrosequencing analysis of KRAS and BRAF mutations in DNA from 1 mm formalin-fixed paraffin-embedded tumour tissue cores taken from areas with >90% tumour cells as previously described [16]. In brief, genomic DNA was extracted from tumour tissue using QIAamp MinElute spin columns (Qiagen) and DNA regions of interest were PCR amplified (Veriti 96 Well Fast Thermal Cycler, Applied Biosystems Inc., Foster City CA). KRAS codons 12 and 13 were analysed using therascreen KRAS Pyro Kit (Qiagen). Analysis of BRAF mutation hotspots in codons 600 and 601 was performed using previously published PCR primers [29] and a novel BRAF sequencing primer (5′- TGATTTTGGTCTAGCTACA-3′) which was designed using the PyroMark Assay Design 2.0 software (Qiagen). All samples with a potential low-level mutation were re-analysed. In the analysis, KRAS is categorised into KRAS wild type (0) or KRAS mutated (1), and BRAF as wild type (0) or mutated (1).

### Statistical methods

Anthropometric measurements were divided into tertiles. Separate tertiles were also calculated for men and women. A Cox proportional hazards analysis was used in order to calculate relative risks of different anthropometric factors and subgroups of CRC defined by KRAS and BRAF mutation status, overall, and stratified for sex. This yielded hazard ratios (HR) with a 95% confidence interval. Follow-up time was defined as time from baseline to diagnosis, death or end of follow-up 31 December 2009. Median time from baseline until diagnosis was 8.6 (SD = 4.3) years in all cases; 8.9 (SD = 4.4) years in men and 8.4 (SD = 4.3) years in women. The proportional hazards assumption was confirmed by a log, - log plot [26]. Potential confounders were included in the multivariate analysis, i.e age (years), educational level (not completed elementary school/elementary school (6–8 years)/“grundskola” (9–10 years)/“studentexamen” (10–12 years)/one year after “studentexamen”/university degree), smoking habits (yes regularly, yes occasionally, former smoker, never smoker), alcohol consumption (g/day), and sex (in the overall analysis). The confounders were chosen on the base of already established and potential risk factors of CRC [27]–[31]. Trend was calculated as linear trend over tertiles. Missing category was not included in the trend analysis.

A two-tailed p-value less than 0.05 was regarded as statistically significant. Chi square test was applied for assessment of the distribution of investigative factors according to baseline characteristics. A case-to-case analysis examined the heterogeneity between different tumour subgroups regarding their association to anthropometric factors using an unconditional logistic regression model. In order to assess any potential interaction between each anthropometric factor and sex, an interaction term was introduced in the logistic regression model. P-values <0.05 were considered statistically significant.

All statistical analyses were conducted using IBM SPSS statistics 20.0 (SPSS Inc., Chicago, IL, USA).

## Results

### Distribution of risk factors in cases and controls, and in strata according to KRAS and BRAF mutation status

Distribution of well-established risk factors in CRC cases and rest of cohort, as well as distribution of KRAS and BRAF mutations in cases, are shown in Table 1. Compared to rest of cohort, CRC cases were slightly older (p<0.001 for both men and women), of higher weight (p = 0.014 for men and p = 0.008 for women), had a higher bodyfat percentage (p = 0.019 for men and p<0.001 for women), a higher waist circumference (p<0.001 for men and p = 0.001 for women), a higher hip circumference (p<0.001 for both sexes), a higher WHR for men (p = 0.021), and a higher BMI (p = <0.001 for men and p = 0.001 for women). Among women, cases had a higher level of education (p = 0.009) and a lower intake of alcohol (p = 0.001) than rest of cohort. Smoking status did not differ between cases and rest of cohort. Of note, while the proportion of current smokers was similar in both sexes, the proportion of former smokers was higher (51.8%) in men than in women.

**Table 1:** Distribution of risk factors in cases and rest of cohort

Characteristics	Rest of cohort	CRC cases	*p*	KRAS wild type	KRAS mutated	*p*	BRAF wild type	BRAF mutated	*p*
n (%)	27514	584		314(63.6)	180(36.4)		423(85.6)	71(14.4)	
**Sex** (%)			*<0.001*			*0.985*			*0.048*
male	39.2	47.9		148(47.1)	85(47.2)		208(88.9)	26(11.1)	
female	60.8	52.1		166(52.9)	95(52.8)		215(82.7)	45(17.3)	
**Age at baseline (years)**									
overall	58.0	61.8	*<0.001*	62.2	61.8	*0.457*	61.8	63.4	*0.044*
male	59.2	61.7	*<0.001*	62.2	61.0	*0.183*	61.6	63.3	*0.209*
female	57.3	62.1	*<0.001*	62.2	62.5	*0.661*	61.9	63.8	*0.174*
**Smoking (%)**			*0.092*			*0.425*			*0.366*
regularly	23.8	21.2		62(19.7)	39(21.7)		84(19.9)	17(23.9)	
occasionally	4.5	3.4		7(2.2)	7(3.8)		14(3.3)	0(0)	
former smoker	33.7	39.7		132(42.0)	64(35.6)		167(39.5)	30(42.3)	
never smoker	37.9	35.6		113(36.0)	70(38.9)		158(37.4)	24(33.8)	
**Smoking male (%)**			*0.104*			*0.348*			*0.457*
regularly	23.9	18.9		23(15.4)	19(10.6)		35(16.7)	7(26.9)	
occasionally	4.8	4.3		3(2.0)	4(2.2)		7(3.3)	0(0)	
former smoker	43.0	51.8		83(56.1)	42(23.3)		112(53.8)	14(53.8)	
never smoker	28.2	25.0		39(26.2)	20(11.1)		54(25.7)	5(19.2)	
**Smoking female (%)**			*0.652*			*0.571*			*0.373*
regularly	23.8	23.3		39(23.4)	20(11.1)		49(22.6)	10(22.2)	
occasionally	4.3	2.6		4(2.4)	3(1.7)		7(3.2)	0(0)	
former smoker	27.7	28.6		49(29.3)	22(12.2)		55(25.3)	16(35.6)	
never smoker	44.2	45.4		74(44.6)	50(27.8)		104(48.4)	19(42.2)	
**Level of education (%)**			*0.023*			*0.429*			*0.409*
not completed	0.8	0.9		3(1.0)	1(0.6)		2(0.4)	2(2.8)	
6–8 years	41.0	49.1		167(53.5)	85(47.5)		214(51.0)	39(54.9)	
9–10 years	26.2	22.4		71(22.8)	37(20.7)		93(22.1)	15(21.1)	
10–12 years	8.9	8.7		25(8.0)	18(10.1)		37(8.8)	5(7.0)	
1 year university	8.7	7.7		22(7.1)	16(8.9)		33(7.9)	5(7.0)	
university degree	14.3	10.8		24(7.7)	22(12.3)		41(9.8)	5(7.0)	
**Level of education male (%)**			*0.912*			*0.793*			*0.042*
not completed	0.8	0.7		1(0.7)	0(0)		0	1(3.8)	
6–8 years	45.1	48.9		78(52.7)	40(22.2)		109(52.4)	10(38.5)	
9–10 years	19.6	19.6		30(20.1)	16(8.9)		42(20.2)	4(15.4)	
10–12 years	11.9	10.4		15(10.1)	10(5.6)		20(9.5)	5(19.2)	
1 year university	9.3	6.7		9(6.0)	6(3.3)		13(6.2)	2(7.7)	
university degree	13.2	13.6		15(10.1)	13(7.2)		24(11.4)	4(15.4)	
**Level of education female (%)**			*0.009*			*0.688*			*0.167*
not completed	0.8	1.0		2(1.2)	1(0.6)		2(0.9)	1(2.2)	
6–8 years	38.5	49.8		89(53.9)	45(0.25)		105(49.5)	29(64.4)	
9–10 years	30.5	24.9		41(24.8)	21(11.7)		51(23.8)	11(24.4)	
10–12 years	7.0	7.3		10(6.1)	8(4.4)		17(7.9)	0(0)	
1 year university	8.4	8.6		13(7.9)	10(5.6)		20(9.4)	3(6.7)	
university degree	15.1	8.3		9(5.5)	9(5.0)		17(7.9)	1(2.2)	
**Alcohol (g/day)**									
overall	10.7	10.8	*0.404*	10.4	11.2	*0.286*	11.2	7.8	*0.052*
male	15.5	15.7	*0.793*	16.3	15.6	*0.925*	16.3	14.2	*0.842*
female	7.7	6.2	*0.001*	5.1	7.3	*0.134*	6.3	4.0	*0.112*
**Height (cm)**									
overall	168.6	169.5	*0.020*	169.4	169.6	*0.792*	169.8	167.5	*0.052*
male	176.4	176.3	*0.400*	176.3	177.2	*0.108*	176.7	175.9	*0.443*
female	163.6	163.3	*0.220*	163.2	162.8	*0.632*	163.2	162.6	*0.432*
**Weight (kg)**									
overall	73.4	76.6	*<0.001*	76.4	77.6	*0.481*	77.4	73.8	*0.075*
male	81.7	83.8	*0.014*	83.3	86.0	*0.173*	84.7	81.2	*0.181*
female	68.0	70.0	*0.008*	70.3	70.0	*0.725*	70.3	69.6	*0.913*
**Bodyfat %**									
overall	26.8	26.9	*0.974*	27.0	27.3	*0.607*	26.9	27.7	*0.372*
male	20.8	21.6	*0.019*	21.2	21.7	*0.676*	21.6	20.0	*0.199*
female	30.2	31.9	*<0.001*	32.0	32.3	*0.595*	32.1	32.1	*0.955*
**Hip (cm)**									
overall	98.4	100.7	*<0.001*	100.8	101.1	*0.617*	101.0	99.9	*0.353*
male	99.4	101.2	*<0.001*	101.2	101.9	*0.517*	101.8	99.5	*0.162*
female	98.0	100.2	*<0.001*	100.4	100.3	*0.984*	100.4	100.2	*0.958*
**Waist (cm)**									
overall	84.1	87.9	*<0.001*	87.4	88.9	*0.224*	88.5	84.8	*0.038*
male	94.0	96.3	*<0.001*	96.0	97.6	*0.334*	97.0	93.6	*0.107*
female	78.2	80.1	*0.001*	79.8	81.0	*0.341*	80.3	79.7	*0.898*
**WHR (cm/cm)**									
overall	0.85	0.87	*<0.001*	0.87	0.88	*0.184*	0.87	0.85	*0.036*
male	0.94	0.95	*0.021*	0.95	0.96	*0.323*	0.95	0.94	*0.349*
female	0.80	0.80	*0.525*	0.79	0.81	*0.062*	0.80	0.80	*0.808*
**BMI (kg/m^2^)**									
overall	25.7	26.6	*<0.001*	26.6	26.9	*0.374*	26.7	26.3	*0.286*
male	26.2	26.9	*<0.001*	26.8	27.4	*0.185*	27.1	26.3	*0.204*
female	25.4	26.3	*0.001*	26.4	26.4	*0.946*	26.4	26.3	*0.935*

KRAS and BRAF mutations were successfully evaluated in 494 (84.6%) cases. A total number of 314 (63.7%) tumours were KRAS wild type and 180 (36.4%) were KRAS-mutated. Further, 423 (85.6%) of the tumours were BRAF wild type, and 71 (14.4%) were BRAF-mutated. KRAS and BRAF mutations were mutually exclusive. BRAF mutation was significantly associated with female sex and with higher age overall.

### CRC risk according to KRAS and BRAF mutation status in the entire cohort

Associations of anthropometric factors with KRAS and BRAF mutation status of the tumours in the entire cohort are shown in Table 2. High weight, bodyfat percentage, hip, waist, WHR and BMI were associated with an increased risk of KRAS-mutated CRC (p_trend_ **= **0.006, p_trend_ **= **0.007, p_trend_ **= **0.004, p_trend_ **= **0.004, p_trend_ **= **0.041, p_trend_ **= **<0.001), and BMI was also associated with an increased risk of KRAS wild type tumours (p_trend_ **= **0.046). Interaction analysis revealed a significant difference between sexes for BMI and risk of KRAS-mutated tumours (p_interaction_ = 0.044).

**Table 2:** Hazard ratio of overall CRC risk defined by KRAS and BRAF mutation status

		KRAS wild-type		KRAS mutated		BRAF wild-type		BRAF mutated	
Anthropometric factor	tertile	n = 314	HR	n = 180	HR	n = 422	HR	n = 71	HR
Height (kg)	1(<164)	90	1.00	55	1.00	117	1.00	27	1.00
	2(≥164-<172)	98	1.10(0.81–1.50)	50	0.94(0.60–1.48)	127	1.06(0.81–1.39)	21	0.94(0.52–1.72)
	3(≥172)	126	1.25(0.84–1.87)	74	0.95(0.53–1.71)	178	1.21(0.85–1.71)	23	1.19(0.52–2.76)
	p trend		0.274		0.846		0.295		0.763
Weight (kg)	1(<67)	84	1.00	46	1.00	107	1.00	23	1.00
	2(≥67-<79)	104	1.11(0.82–1.49)	53	1.21(0.76–1.92)	133	1.10(0.85–1.44)	24	1.09(0.60–1.98)
	3(≥79)	126	1.30(0.95–1.79)	80	1.87(1.17–2.99)	182	1.44(1.09–1.90)	24	1.17(0.61–2.23)
	p trend		0.099		0.006		0.007		0.640
Bodyfat%	1(<23)	95	1.00	54	1.00	131	1.00	19	1.00
	2(≥23-<31)	106	1.26(0.92–1.74)	55	1.42(0.89–2.26)	137	1.31(0.99–1.71)	24	1.06(0.51–2.21)
	3(≥31)	111	1.43(0.96–2.13)	70	2.20(1.23–3.91)	152	1.73(1.23–2.43)	28	1.00(0.42–2.37)
	p trend		0.087		0.007		0.002		0.966
Hip (cm)	1(<77)	84	1.00	35	1.00	98	1.00	21	1.00
	2(≥77-<90)	143	0.90(0.66–1.22)	87	1.48(0.92–2.38)	126	1.13(0.87–1.48)	18	0.81(0.43–1.54)
	3(≥90)	87	1.20(0.91–1.59)	58	1.89(1.21–2.95)	198	1.46(1.14–1.87)	32	1.09(0.62–1.91)
	p trend		0.123		0.004		0.002		0.695
Waist (cm)	1(<95)	79	1.00	38	1.00	97	1.00	20	1.00
	2(≥95-<101)	98	1.01(0.74–1.39)	54	1.06(0.67–1.71)	124	1.07(0.80–1.41)	28	1.18(0.65–2.16)
	3(≥101)	137	1.29(0.91–1.83)	87	1.98(1.20–3.28)	201	1.60(1.18–2.16)	23	0.99(0.48–2.02)
	p trend		0.120		0.004		0.001		0.962
WHR (cm/cm)	1(<0.80)	100	1.00	44	1.00	118	1.00	26	1.00
	2(≥0.80-<0.90)	91	0.85(0.62–1.15)	62	1.29(0.82–2.01)	128	1.04(0.78–1.37)	24	0.88(0.48–1.58)
	3(≥0.90)	123	0.93(0.60–1.45)	73	1.98(1.04–3.74)	176	1.24(0.85–1.82)	21	0.80(0.32–2.01)
	p trend		0.647		0.041		0.291		0.609
BMI (kg/m^2^)	1(<23.8)	81	1.00	42	1.00	103	1.00	20	1.00
	2(≥23.8<27.1)	106	1.05(0.78–1.40)	54	1.04(0.65–1.66)	136	1.07(0.82–1.39)	24	1.01(0.55–1.85)
	3(≥27.1)	127	1.32(0.99–1.76)	83	2.11(1.38–3.22)	183	1.53(1.20–1.97)	21	1.15(0.64–2.08)
	p trend		0.046		<0.001¶		<0.001		0.627

Adjusted for age, sex, level of education, smoking habits and alcohol consumption. ¶ significant interaction with sex.

Increased weight, bodyfat percentage, hip, waist and BMI were associated with risk of BRAF wild type tumours (p_trend_ **= **0.007, p_trend_ **= **0.002, p_trend_ **= **0.002, p_trend_ **= **0.001, p_trend_ **= **<0.001). None of the anthropometric factors were associated with BRAF-mutated CRC.

### CRC risk according to KRAS and BRAF mutation status in men

Associations of anthropometric factors with KRAS and BRAF mutation status of CRC tumours in men are shown in Table 3. While elevated height was not associated with CRC risk according to KRAS or BRAF mutation status, high weight was significantly associated with KRAS-mutated and BRAF wild type CRC (p_trend_ **= **0.004 and p_trend_ **= **0.001). Moreover, bodyfat percentage was significantly associated with BRAF wild type CRC. High waist- and hip measures were associated with an increased risk of KRAS wild type tumours (p_trend_ **= **0.045 and p_trend_ **= **0.043), KRAS-mutated tumours (p_trend_ **= **0.007 and p_trend_ **= **<0.001), as well as BRAF wild type tumours (p_trend_ **= **<0.001 and p_trend_ **= **<0.001). Moreover, high WHR and BMI were positively associated with an increased risk of KRAS-mutated and BRAF wild type CRC (p_trend_ **= **<0.001 and p_trend_ **= **0.001).

**Table 3:** Hazard ratio of CRC risk defined by KRAS and BRAF mutation status in men

		KRAS wild-type		KRAS mutated		BRAF wild-type		BRAF mutated	
Anthropometric factor	tertile	n = 148	HR	n = 85	HR	n = 208	HR	n = 26	HR
Height (cm)	1(<173)	37	1.00	21	1.00	52	1.00	6	1.00
	2(≥173-<179)	63	1.50(1.00–2.26)	26	1.06(0.59–1.89)	76	1.26(0.89–1.80)	14	2.18(0.83–5.72)
	3(≥179)	48	1.18(0.76–1.83)	38	1.50(0.87–2.59)	80	1.35(0.94–1.93)	6	0.92(0.29–2.93)
	p trend		0.513		0.126		0.110		0.875
Weight (kg)	1(<76)	43	1.00	18	1.00	53	1.00	8	1.00
	2(≥76-<86)	41	0.80(0.52–1.24)	27	1.30(0.72–2.29)	59	0.95(0.65–1.37)	9	1.02(0.39–2.67)
	3(≥86)	64	1.37(0.93–2.03)	40	2.10(1.25–3.87)	96	1.69(1.20–2.38)	10	1.18(0.44–3.11)
	p trend		0.083		0.004		0.001		0.742
Bodyfat%	1(<18)	31	1.00	16	1.00	39	1.00	8	1.00
	2(≥18-<22)	45	1.07(0.68–1.69)	28	1.30(0.71–2.41)	66	1.24(0.84–1.85)	8	0.79(0.30–2.12
	3(≥22)	70	1.39(0.91–2.12)	41	1.66(0.93–2.97)	101	1.60(1.11–2.33)	10	0.87(0.34–2.23)
	p trend		0.101		0.078		0.009		0.797
Hip (cm)	1(<96)	31	1.00	12	1.00	35	1.00	8	1.00
	2(≥96-<102)	53	1.23(0.79–1.92)	35	2.22(1.15–4.29)	79	1.65(1.11–2.47)	9	0.84(0.32–2.20)
	3(≥102)	64	1.54(1.00–2.38)	38	2.55(1.32–4.92)	94	2.06(1.39–3.05)	9	0.87(0.33–2.31)
	p trend		0.045		0.007		<0.001		0.795
Waist (cm)	1(<89)	38	1.00	14	1.00	42	1.00	10	1.00
	2(≥89-<97)	40	0.90(0.57–1.40)	23	1.45(0.74–2.82)	58	1.18(0.80–1.76)	5	0.45(0.15–1.32)
	3(≥97)	70	1.45(0.97–2.16)	48	2.92(1.60–5.33)	108	2.06(1.44–2.95)	11	0.96(0.40–2.30)
	p trend		0.043		<0.001		<0.001		0.972
WHR (cm/cm)	1(<0.80)	44	1.00	23	1.00	59	1.00	8	1.00
	2(≥0.80-<0.90)	51	1.15(0.77–1.72)	24	1.06(0.60–1.89)	64	1.08(0.76–1.54)	11	1.44(0.78–3.59)
	3(≥0.90)	53	1.45(0.97–2.17)	38	2.13(1.26–3.61)	85	1.76(1.25–2.46)	7	1.20(0.43–3.34)
	p trend		0.073		0.004		0.001		0.697
BMI (kg/m^2^)	1(<24.7)	46	1.00	19	1.00	55	1.00	10	1.00
	2(≥24.7<27.5)	43	0.81(0.53–1.23)	24	1.18(0.64–2.16)	60	0.96(0.67–1.39)	7	0.66(0.25–1.74)
	3(≥27.5)	59	1.21(0.82–1.80)	42	2.44(1.41–4.23)	93	1.67(1.19–2.35)	9	0.97(0.39–2.44)
	p trend		0.287		0.001		0.001		0.939

Adjusted for age, level of education, smoking habits and alcohol consumption.

Due to the comparatively small subgroup of male subjects with BRAF-mutated tumours, all analyses were also performed with continuous anthropometric variables, which did not alter the significant associations (data not shown).

### CRC risk according to KRAS and BRAF mutation status in women

As shown in Table 4, a high WHR was significantly associated with an increased risk of KRAS-mutated CRC (p_trend_ **= **0.046) as compared to KRAS wild type tumours in women, with a significant heterogeneity in the highest tertile (p = 0.032). Moreover, high bodyfat percentage was significantly associated with an increased risk of BRAF wild type tumours (p_trend_ **= **0.032).

**Table 4:** Hazard ratio of CRC risk defined by KRAS and BRAF mutation status in women.

		KRAS wild-type		KRAS mutated		BRAF wild-type		BRAF mutated	
Anthropometric factor	tertile	n = 166	HR	n = 94	HR	n = 214	HR	n = 45	HR
Height (kg)	1(<161)	60	1.00	28	1.00	68	1.00	19	1.00
	2(≥161-<166)	47	0.88(0.60–1.29)	38	1.44(0.88–2.36)	73	1.15(0.83–1.61)	12	0.76(0.37–1.57)
	3(≥166)	59	1.17(0.80–1.70)	28	1.12(0.65–1.93)	73	1.19(0.84–1.68)	14	1.07(0.52–2.18)
	p trend		0.443		0.658		0.318		0.926
Weight (kg)	1(<62)	36	1.00	23	1.00	48	1.00	11	1.00
	2(≥62-<71)	62	1.47(0.97–2.22)	36	1.36(0.80–2.29)	81	1.44(1.01–2.07)	17	1.33(0.62–2.86)
	3(≥71)	68	1.51(1.00–2.27)	35	1.22(0.72–2.08)	85		17	1.13(0.53–2.44)
	p trend		0.061		0.508		0.057		0.806
Bodyfat%	1(<29)	32	1.00	22	1.00	43	1.00	11	1.00
	2(≥29-<33)	56	1.38(0.89–2.15)	23	0.90(0.50–1.62)	68	1.31(0.89–1.93)	11	0.75(0.32–1.73)
	3(≥33)	78	1.41(0.92–2.15)	49	1.42(0.84–2.38)	103	1.50(1.04–2.16)	21	1.01(0.49–2.12)
	p trend		0.144		0.125		0.032		0.834
Hip (cm)	1(<93)	37	1.00	22	1.00	47	1.00	12	1.00
	2(≥93-<101)	59	1.10(0.72–1.67)	30	0.97(0.56–1.69)	77	1.15(0.80–1.67)	12	0.66(0.30–1.48)
	3(≥101)	70	1.21(0.81–1.83)	42	1.28(0.75–2.17)	90	1.30(0.90–1.86)	21	0.95(0.46–1.97)
	p trend		0.343		0.309		0.159		0.951
Waist (cm)	1(<72)	39	1.00	22	1.00	50	1.00	11	1.00
	2(≥72-<81)	62	1.09(0.73–1.63)	31	1.02(0.59–1.76)	80	1.13(0.79–1.61)	13	0.77(0.35–1.73)
	3(≥81)	65	1.11(0.74–1.66)	41	1.32(0.77–.2.24)	84	1.17(0.83–1.70)	21	1.06(0.51–2.24)
	p trend		0.645		0.268		0.361		0.745
WHR (cm/cm)	1(<0.77)	61	1.00	25	1.00	75	1.00	11	1.00
	2(≥0.77-<0.81)	61	0.93(0.65–1.34)	33	1.19(0.71–2.01)	72	0.90(0.65–1.24)	22	1.74(0.84–3.59)
	3(≥0.81)	44	0.80(0.54(1.19)	36	1.68(1.00–2.81)*	67	1.05(0.75–1.46)	12	1.06(0.46–2.41)
	p trend		0.270		0.046		0.822		0.920
BMI (kg/m^2^)	1(<23.2)	35	1.00	25	1.00	52	1.00	8	1.00
	2(≥23.2-<26.6)	67	1.54(1.02(2.33)	30	1.04(0.61–1.77)	75	1.21(0.84–1.73)	22	2.07(0.92–4.68)
	3(≥26.6)	64	1.40(0.92–2.14)	39	1.28(0.76–2.15)	87	1.38(0.97–1.96)	15	1.20(0.50–2.87)
	p trend		0.162		0.329		0.077		0.939

Adjusted for age, sex, level of education, smoking habits and alcohol consumption. * significant p for heterogeneity.

## Discussion

In this prospective cohort study, we have investigated the relationship between obesity, measured as several anthropometric factors, and risk of CRC according to KRAS and BRAF mutation status of the tumours, with particular reference to potential sex differences. In the overall analysis, significant associations were found between most anthropometric factors and risk of KRAS-mutated and BRAF wild type tumours, whereas only BMI was associated with KRAS wild type tumours, and none of the anthropometric factors were associated with risk of BRAF-mutated CRC. Further analyses stratified for sex revealed that these associations were similar in the male but not in the female subcohort. Of note, a significant interaction was only found between sex and BMI with respect to risk of KRAS-mutated tumours, and heterogeneity analysis was only significant for the highest tertile of WHR in relation to risk of KRAS-mutated compared to KRAS wild type tumours among women.

Despite a growing awareness of the molecular heterogeneity of CRC, comparatively few studies have evaluated CRC risk in relation to its various molecular phenotypes [32]–[35]. To our knowledge, the relationship between obesity, KRAS mutation status and CRC risk has only been investigated in a few previous studies. Slattery et al have studied the associations between diet, physical activity, KRAS mutation status and risk of rectal cancer, demonstrating a reduced risk of KRAS-mutated rectal cancer with a high intake of vegetables, fibre and a high level of physical activity. Moreover, BMI was not associated with overall risk of rectal cancer, nor with specific molecular subtypes thereof [19]. In another study, Slattery et al have shown that men, but not women, with low levels of physical activity were more likely to have KRAS-mutated colon cancer, and that KRAS-mutated tumours were more common in women with a higher BMI [36]. The results from the present study appear to be somewhat different in that a high BMI was found to be associated with risk of KRAS-mutated tumours in men but not in women, with a significant interaction of sex with BMI regarding this risk.

The prognostic role of KRAS mutation has been more extensively investigated, with diverging results [7], [8], [37].

Of note, in the here examined cohort, it has recently been shown that mutation in KRAS codon 13, but not in codon 12, was associated with a significantly reduced cancer specific survival which is in line with other previous publications [16], [38], [39] [40], and most previous studies have not considered the prognostic value of specific mutations [7]–[9], [37], [41]. Furthermore, KRAS codon 13 mutation was found to be significantly associated with metastatic disease, and codon 12 mutation with mucinous tumour type, indicating that specific KRAS mutations have different impact on protein functionality, and hence influence clinical outcome in CRC patients differently [16]. Moreover, KRAS codon 13 mutation was also found to be associated with poor prognosis in women, but not in men [16]. In light of these findings, it will also be relevant to study whether the associations of anthropometric factors with risk of CRC defined by specific KRAS mutations may differ between sexes. This will however require a larger sample size, as KRAS codon 13 mutations are less frequent than KRAS codon 12 mutations.

In the here studied cohort, BRAF mutation has previously been demonstrated to be an independent factor of poor prognosis in men, but not in women, in particular in MSS tumours [24]. It is well established that BRAF mutation, in contrast to KRAS mutation, is associated with MSI [9], [15], [41] and female sex [41], [42]. MSI has generally been associated with good prognosis in most, but not all, studies [43], [44]. On the other hand, BRAF mutation is generally associated with an inferior patient survival [9], [14], [45]. We have recently presented data showing that obesity was not associated with MSI tumours in any of the sexes, but that high weight, hip circumference and BMI was significantly associated with MSS CRC in women, and that high waist and hip circumference was significantly associated with MSS CRC in men [46]. The findings from the present study demonstrate a significant association of obesity with BRAF wild type tumours, being particularly evident in men. This suggests that obesity is more related to MSS tumours, and to tumours lacking BRAF mutation. Two previous studies have investigated the association between BMI and BRAF status in CRC tumours. In a case-control study, Slattery et al. reported that obesity was not associated with BRAF-mutated tumours, but associations with BRAF wild type tumours were not reported [47]. Further, Hughes et al presented data showing that BMI and waist measurements were strong risk factors for BRAF wild type tumours, which is consistent with our findings [20].

While it is well documented that body size influences CRC risk, also with differences regarding sex, location, and tumour stage [48], the exact biologic mechanisms underlying the association between obesity and increased risk of CRC are not fully understood. A large number of studies have shown an increased risk of CRC in men, but not in women, and the reason for this sex difference remains unclear, but is most probable due to hormonal factors [49], [50]. BMI has been the most commonly used measurement of obesity, which may not be ideal because of the changes in physiologic functions that may depend on differences in adipose tissue distribution. A few prospective studies have examined the association of body fat distribution, reflected as waist- and hip circumference, and CRC risk [51]–[53], and available epidemiologic evidence suggests that abdominal obesity (high waist circumference and waist-hip-ratio) may be more predictive of CRC risk than overall obesity [52]–[54]. Increased bodyweight has been suggested to be more closely related to abdominal obesity in men, and to gluteofemoral obesity in women [55] and central adiposity is thought to be a better predictor of CRC risk than BMI [54].

General strengths of this study are the relatively large number of CRC cases, and the prospective design. However, a statistical issue to be addressed is the rather small numbers emerging in the subgroup analyses and, subsequently, limited statistical power with a potential risk of true associations not being detected, i.e. a type II error. Risk estimates in small groups often result in wide confidence intervals and, consequently, poor precision. Therefore, such risk estimates will need careful interpretation and validation in additional patient cohorts. Moreover, it should also be pointed out that a relatively large number of comparisons have been performed, which may result in a type I error, i.e. the null hypothesis being rejected when it is actually true.

The validity of the anthropometric measurements is another methodological aspect, as there may be a potential inter-observer variation. Recommendations for the nurses performing baseline examinations described how participants should be dressed, in which position the participants should be examined, and location for the estimation of waist- and hip measurements. We therefore consider the risk of misclassification of anthropometric measurements to be low. In contrast, most previous studies have used self- reported anthropometric measures. As anthropometric data was assessed only at baseline, it is further possible that some individuals have gained and some have lost weight. Such a misclassification is likely to lead to an attenuation of risks and, if anything, observed risks may be underestimated.

It is also possible that participation in the MDCS was associated with body constitution, which may have lead to a potential selection bias. In a previous paper, Manjer et al compared BMI in the MDCS population in relation to the background population, and found an equal distribution of overweight and obesity [56].

Screening for CRC will most probably increase rapidly in westernized countries, and it is therefore a great challenge to identify individuals at risk of developing CRC. Detection and removal of adenomas are feasible by endoscopic techniques, but the majority of adenomas will not progress to cancer. Thus, defining the “adenoma at risk”, and consequently, the “patient at risk”, in relation to specific molecular subgroups of CRC, is a major research challenge. Moreover, given that the global prevalence of overweight and obesity continues to rise, it is of great importance to invest in primary prevention.

## Conclusions

In conclusion, the results from this prospective study provide further support to the accumulating evidence of the influence of lifestyle factors and sex on different pathways of colorectal carcinogenesis, defined by KRAS and BRAF mutation status of the tumours. These findings need to be confirmed in additional molecular pathological epidemiology studies, in order to gain further insight into the interplay between lifestyle and colorectal carcinogenesis, with the ultimate goal to develop improved strategies for individualized prevention of the disease.
